# Comprehensive analysis of prognostic biomarkers in lung adenocarcinoma based on aberrant lncRNA–miRNA–mRNA networks and Cox regression models

**DOI:** 10.1042/BSR20191554

**Published:** 2020-01-31

**Authors:** Yan Yao, Tingting Zhang, Lingyu Qi, Ruijuan Liu, Gongxi Liu, Jia Wang, Qi Song, Changgang Sun

**Affiliations:** 1 Clinical Medical Colleges, Weifang Medical University. Postal Addresses: NO.7166, Baotong western street, WeiFang, ShanDong Province, P.R. China; 2 College of First Clinical Medicine, Shandong University of Traditional Chinese Medicine. Postal Addresses: NO.16369, Jingshi Road, Jinan, ShanDong Province, P.R. China; 3 Department of Oncology, Weifang Traditional Chinese Hospital. NO.1055, WeiZhouRoad, WeiFang, ShanDong Province, P.R.China; 4 College of Traditional Chinese Medicine, Shandong University of Traditional Chinese Medicine, Postal addresses: NO.16369, Jingshi Road, Jinan, ShanDong Province, P.R. China; 5 Innovative Institute of Chinese Medicine and Pharmacy, Shandong University of Traditional Chinese Medicine, Jinan 250014, Shandong, PR China

**Keywords:** Competing endogenous RNA network, Cox regression, Lung adenocarcinoma, Overall survival, Prognostic biomarker

## Abstract

Lung adenocarcinoma (LUAD) is the leading cause of cancer-related death worldwide, and its underlying mechanism remains unclear. Accumulating evidence has highlighted that long non-coding RNA (lncRNA) acts as competitive endogenous RNA (ceRNA) and plays an important role in the occurrence and development of LUAD. Here, we comprehensively analyzed and provided an overview of the lncRNAs, miRNAs, and mRNAs associated with LUAD from The Cancer Genome Atlas (TCGA) database. Then, differentially expressed lncRNAs (DElncRNA), miRNAs (DEmiRNA), and mRNAs (DEmRNA) were used to construct a lncRNA–miRNA–mRNA regulatory network according to interaction information from miRcode, TargetScan, miRTarBase, and miRDB. Finally, the RNAs of the network were analyzed for survival and submitted for Cox regression analysis to construct prognostic indicators. A total of 1123 DElncRNAs, 95 DEmiRNAs, and 2296 DEmRNAs were identified (|log_2_FoldChange| (FC) > 2 and false discovery rate (FDR) or adjusted *P* value < 0.01). The ceRNA network was established based on this and included 102 lncRNAs, 19 miRNAs, and 33 mRNAs. The DEmRNAs in the ceRNA network were found to be enriched in various cancer-related biological processes and pathways. We detected 22 lncRNAs, 12 mRNAs, and 1 miRNA in the ceRNA network that were significantly associated with the overall survival of patients with LUAD (*P* < 0.05). We established three prognostic prediction models and calculated the area under the 1,3,5-year curve (AUC) values of lncRNA, mRNA, and miRNA, respectively. Among them, the prognostic index (PI) of lncRNA showed good predictive ability which was 0.737, 0.702 and 0.671 respectively, and eight lncRNAs can be used as candidate prognostic biomarkers for LUAD. In conclusion, our study provides a new perspective on the prognosis and diagnosis of LUAD on a genome-wide basis, and develops independent prognostic biomarkers for LUAD.

## Introduction

Lung cancer is considered the leading cause of cancer-related deaths worldwide. Lung adenocarcinoma (LUAD) is the most prevalent pathological type of lung cancer; it accounts for approximately 40% of lung cancer cases and the average 5-year survival rate is only 18% [1–3]. Out of the 8.8 million cancer deaths in 2015, 1.69 million were due to lung cancer [4]. About 60–85% of all lung cancer patients are discovered at advanced stages [5]. Individualized treatment guided by genetic testing results has become the main treatment for LUAD. However, the essential biomarkers and precise targets for the development and progression of LUAD remain unclear. Such cases underscore the critical need to find more rational, specific, and effective treatments and prognostic targets.

Increasing numbers of studies have indicated that next-generation sequencing techniques may provide new insights into the alteration of gene expression during tumorigenesis and help to better identify clinical biomarkers of cancer [6,7]. The application of high-throughput methods enables researchers to identify RNAs as biomarkers in cancer diagnosis and treatment. The discovery of long non-coding RNA (lncRNA) has dramatically altered our understanding of cancer. Generally, lncRNAs are >200 nucleotides (nt) long with no or limited protein-coding capacity [8]. The latest research shows that lncRNA expression is frequently dysregulated in cancer, and specific lncRNAs are correlated with cancer recurrence, metastasis, and poor prognosis in different kinds of cancer, including LUAD [9,10]. Because of the strong tissue specificity, lncRNAs may be early diagnostic biomarkers for various cancers [11]. However, much still remains unknown about the mechanics and significance of lncRNAs in many aspects of this disease, such as carcinogenesis, development, metastasis, response to anti-cancer treatment, and prognosis. Therefore, the identification of specific lncRNA biomarkers related to the prognosis and diagnosis of LUAC has important clinical significance.

Competing endogenous RNAs (ceRNAs) are proposed as tumor-specific regulatory pathways that affect protein levels [12]. The central concept is that messenger RNA (mRNA) and lncRNA share one or more microRNA (miRNA) response elements (MREs) and may act as natural miRNA sponges that lead to the down-regulation of the intracellular miRNA function [13]. Studies have demonstrated that various types of RNAs, such as mRNA, pseudogenes, lncRNA, circular RNA, and miRNA, can communicate with each other through ceRNA mechanisms, thereby regulating various tumor cells and their microenvironment, and affecting tumor proliferation and migration [14]. The introduction of ceRNA theory has revealed a new mechanism for interaction between RNAs, which has broadened our understanding of lncRNA function [15]. In LUAD, the potential of lncRNA to act as a biomarker for cancer prognosis has been widely reported [16–18]. Nevertheless, much still remains unknown about the ceRNA in LUAD and targets for tumor therapy.

In the present study, we comprehensively analyzed and provided an overview of the lncRNAs, miRNAs, and mRNAs in LUAD from The Cancer Genome Atlas (TCGA) database. Three differentially expressed RNAs were used to construct a lncRNA–miRNA–mRNA regulated ceRNA network. Functional enrichment analyses were performed to reveal and determine the functional roles and underlying mechanisms of the ceRNA network in LUAD. Next, we performed survival analysis, univariate, and multivariate Cox proportional hazard regression analysis of three RNAs in the ceRNA network, and established three new types of RNA models to predict the prognosis of LUAD patients. Through the construction of a ceRNA network and Cox regression models, we aimed to elucidate the regulatory mechanisms of lncRNA in the prognosis of LUAD to identify novel biomarkers that can effectively predict clinical outcomes.

## Materials and methods

### Data resources and pretreatment

Individual lung adenocarcinoma RNA sequencing (RNA-seq) data (level 3) and the corresponding clinical data were obtained from the TCGA database. The TCGA database is a public platform with more than 30 cancer types and clinical pathological information for at least 11,000 patients. It has been widely used by a large number of researchers to explore the genetic basis of tumors through high-throughput sequencing. The RNA-seq data were generated by Illumina HiSeq RNA-seq and Illumina HiSeq miRNA-Seq platforms. Exclusion criteria were set as follows: (1) histological diagnosis negating LUAD; (2) presence of a malignancy other than LUAD; (3) lack of complete clinical data. The three RNA expression profiles were integrated and extracted by using the R bioconductor package TCGABiolinks. Genes were annotated using the Ensembl online database. The present study was in compliance with the publication guidelines provided by TCGA, and the data obtained from TCGA did not require approval from an ethics committee.

### Identification of differentially expressed lncRNAs, miRNAs, and mRNAs

To identify the differentially expressed lncRNAs (DElncRNA), miRNAs (DEmiRNA), and mRNAs (DEmRNA) between the LUAD and the normal samples, the downloaded lncRNA, miRNA, and mRNA data were standardized and differential expression analysis was performed using the edgeR package in R software. Filtering criteria for the differential expression of these three RNAs were as follows: (1) at least 25% of the samples have a gene expression level greater than 2; (2) the original RNA sequencing was normalized using the trimmed mean of *M*-values (TMM) method; (3) differential expression thresholds of |log_2_FoldChange| (FC) > 2 and false discovery rate (FDR) or adjusted *P* value < 0.01. Heat map clustering and a volcano plot of the DElncRNAs, DEmiRNAs, and DEmRNAs were plotted using the R software gplots package.

### Establishment of the lncRNA–miRNA–mRNA ceRNA network

Based on the hypothesis that lncRNA can act as a sponge for miRNA and prevent miRNA from binding to its target genes, and can directly interact with mRNA [19,20], the lncRNA–miRNA–mRNA ceRNA network was constructed by the following steps: (1) the miRcode online tool (http://www.mircode.org) was used to predict the potential miRNAs targeted by DElncRNAs and the lncRNA–miRNA interaction pairs; (2) the miRNA sequences were modified and the names of DEmiRNAs were transformed into human mature miRNA names by using the starBase v2.0 database (http://starbase.sysu.edu.cn) [21]; (3) miRDB (http://www.mirdb.org/) [22], miRTarBase (http://mirtarbase.mbc.nctu.edu.tw/) [23], and TargetScan (http://www.targetscan.org/) [24] were used to predict target genes of the DEmiRNAs and establish the miRNA–mRNA interaction pairs; (4) the online website Venny was used to compare the target genes with DEmRNAs and the target genes that overlapped with DEmRNAs in the present study were selected for further analysis. Finally, combined with the lncRNA–miRNA pairs and miRNA–mRNA pairs, we established the lncRNA–miRNA–mRNA ceRNA network. Cytoscape software version 3.6.1was used to visualize the resulting network.

### Functional enrichment analysis of the ceRNA network

Based on the correlation between the three RNAs, we inferred the potential biological processes and pathways of abnormal expression of RNAs in ceRNA networks by analyzing mRNAs in networks. The Annotate, Visualize, and Integrate Discovery Database (DAVID6.8) (http://david.abcc.ncifcrf.gov/) online tool was used to perform Gene Ontology (GO) and Kyoto Encyclopedia of Genes and Genomes (KEGG) pathway analysis. The results of GO analysis mainly consist of three parts: biological process (BP), cellular component (CC), and molecular function (MF). GO and KEGG terms with *P* < 0.05 were considered statistically significant, and the “Goplot” package in R was used to visualize the results as chord plots.

### Overall survival analysis and construction of the LUAD-specific prognostic model

We utilized the survival package to generate Kaplan–Meier separate survival analysis and log-rank tests for the DElncRNAs, DEmiRNAs, and DEmRNAs in the ceRNA network to determine the overall survival (OS) relationship with LUAD patients in TCGA. A *P* value less than 0.05 was considered as statistically significant. The associations of three types of RNAs with OS were assessed by univariate Cox regression. Only those genes with a *P* < 0.01 were identified as candidate prognosis related biomarkers. Then, those candidate genes were analyzed by multivariate Cox regression to remove any genes that might not be independent factors in prognosis prediction. Finally, based on the multivariate Cox hazards regression model to determine the LUAD independent prognostic factors, the lncRNA, miRNA, and mRNA signature scores were constructed, and the prognostic index (PI) of OS was predicted as follows:

$$Prognostic\;index=Exp_{RNAI}*\beta_{RNAI}+Exp_{RNA2}*\beta_{RNA2}+\;\;\;•\;•\;•\;Exp_{RNAn}*\beta_{RNAn}$$

(where “Exp” denotes the expression level of DERNAs, and “β” is the regression coefficient obtained from the multivariate Cox regression model) [25]. LUAD patients were stratified into high-risk and low-risk groups with a median risk score as a threshold. The survival package in R was used to plot the Kaplan–Meier (K-M) survival curve of the two groups. In addition, using the survival ROC package in R, we constructed time-dependent receiver operating characteristic (ROC) curves within 1,3,5-year and estimated the area under the ROC curve (AUC) for the three RNA models.

## Results

### Patient characteristics and differentially expressed RNAs

**Figure 1:**
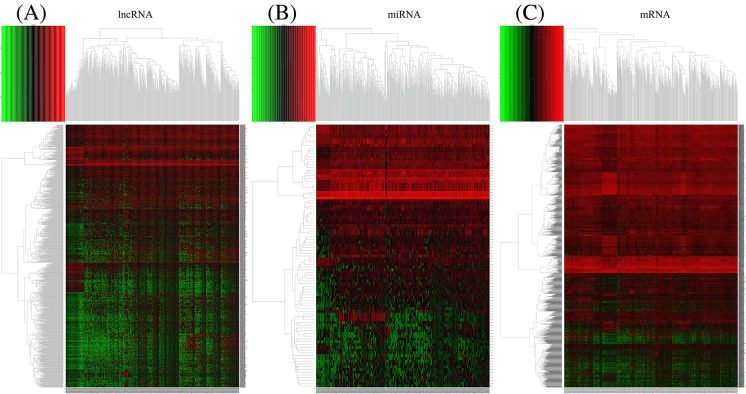
Clustered heat maps of the differentially expressed RNAs in LUAD The rows represent RNAs and columns represent the samples. |log_2_FC| > 2, FDR < 0.01. (**A**–**C**) Differentially expressed lncRNAs, miRNAs, and mRNAs in LUAD. FC: fold change, FDR: false discovery rate, LUAD: lung adenocarcinoma, lncRNAs: long noncoding RNAs, miRNAs: microRNAs, mRNAs: messenger RNAs.

**Figure 2:**
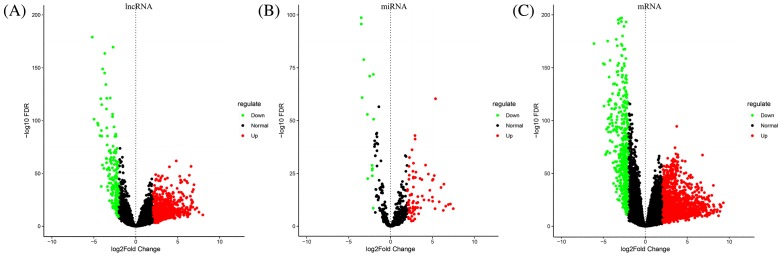
Volcano plot of differentially expressed RNAs in LUAD |log_2_FC| > 2, FDR < 0.01. (**A**–**C**) Differentially expressed lncRNAs, miRNAs, and mRNAs in LUAD. The red dot represents up-regulated RNAs and green dot represents down-regulated RNAs.

The LUAD transcriptome profiling data and the corresponding clinical information were obtained using the R bioconductor package TCGABiolinks. Gene expression quantification data for a total of 594 samples, which included 535 tumor samples and 59 normal samples, and miRNA expression quantification data for 567 samples, which included 521 tumor samples and 46 normal samples. Using the edgeR package in R with the cut-off criteria of |log_2_FC| > 2 and FDR < 0.01, we identified 1123 DElncRNAs (940 up-regulated lncRNAs, 183 down-regulated lncRNAs), 95 DEmiRNAs (81 up-regulated miRNAs, 14 down-regulated miRNAs), and 2296 DEmRNAs (1781 up-regulated mRNAs, and 515 down-regulated mRNAs). The heat map shown in Figure 1 depicted the variable gene expression in the LUAD and normal samples and the volcano plots visually demonstrating the distribution of DElncRNAs, DEmiRNAs, and DEmRNAs were shown in Figure 2.

### Construction of the ceRNA network in lung adenocarcinoma

**Figure 3:**
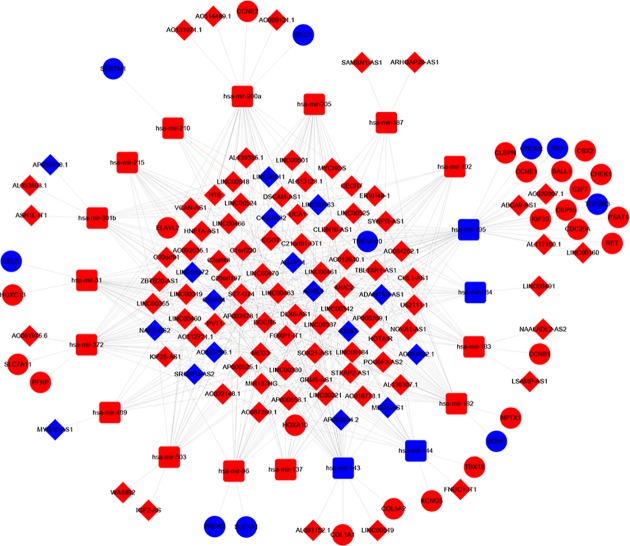
ceRNA networks of lung adenocarcinoma Red represents up-regulation, and blue represents down-regulation. LncRNAs, miRNAs, and mRNAs in the networks are represented as diamonds, round rectangles, and circles, respectively.

According to the relationship between DElncRNAs, DEmiRNAs, and DEmRNAs, we established a lncRNA–miRNA–mRNA ceRNA network in LUAD. (1) The online website miRcode was used to clarify the correlation between miRNAs targeting lncRNAs in the ceRNA cross-talk, the results showed that 102 DElncRNAs could interact with 86 miRNAs, but only 19 out of 86 miRNAs were utilized in relation in our study; (2) using the miRDB, miTarBase, and TargetScan online databases, 19 miRNAs were found to interact with 667 target genes. At the intersection of these 667 target genes and 2296 DEmRNAs, 33 overlapping genes were obtained; (3) based on the DElncRNAs–DEmiRNAs pairs and DEmiRNAs–DEmRNAs pairs, the ceRNA network was constructed and visualized with the Cytoscape software (Figure 3), which comprised of 102 lncRNAs (17 down-regulated, 85 up-regulated), 19 miRNAs (4 down-regulated, 15 up-regulated), and 33 mRNAs (10 down-regulated, 23 up-regulated).

### GO and KEGG enrichment analysis of DEmRNAs in ceRNA

**Figure 4:**
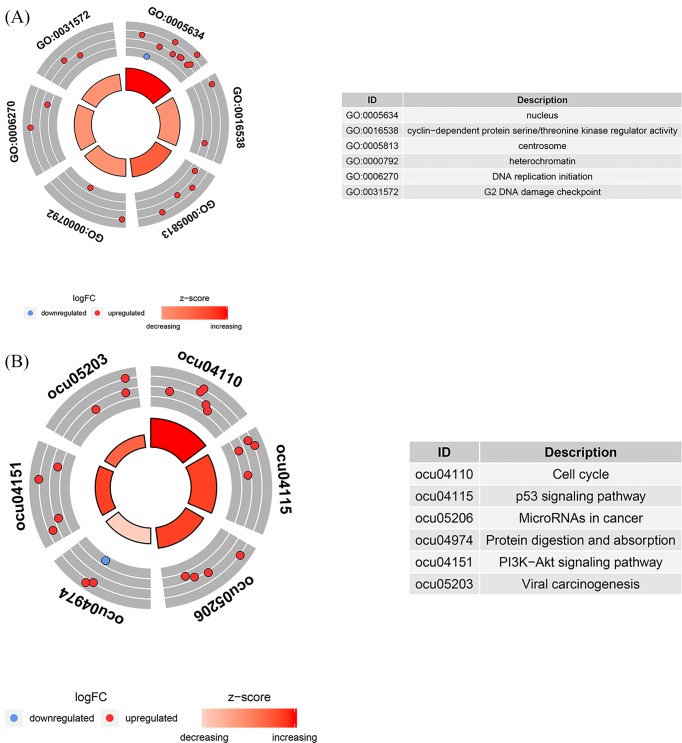
Gene Ontology (GO) analysis and Kyoto Encyclopedia of Genes and Genomes (KEGG) pathways of genes included in the ceRNA network (**A**) Chord plot display of the relationship between genes and GO analysis. (**B**) Chord plot display of the relationship between genes and KEGG pathways.

To better understand the underlying mechanisms of ceRNA networks, we performed functional enrichment analysis of mRNAs in the network with the DAVID database. The GO enrichment analysis showed that six significant terms are involved in LUAD (*P* < 0.05) (Figure 4A). Among them, the BP enrichment process for GO analysis mainly included DNA replication initiation and G2 DNA damage checkpoint. MF enrichment revealed mainly cyclin-dependent protein serine/threonine kinase regulator activity, and CC enrichment mainly included nucleus, centrosome, and heterochromatin. KEGG pathway analysis focused on biological pathways, and a total of five pathways were found to be significantly enriched; cell cycle, p53 signaling pathway, microRNAs in cancer, protein digestion and absorption, and PI3K-Akt signaling pathway. All mentioned pathways are exhibited in Figure 4B.

### Survival analysis and establishment of the three RNA type prognostic model

**Figure 5:**
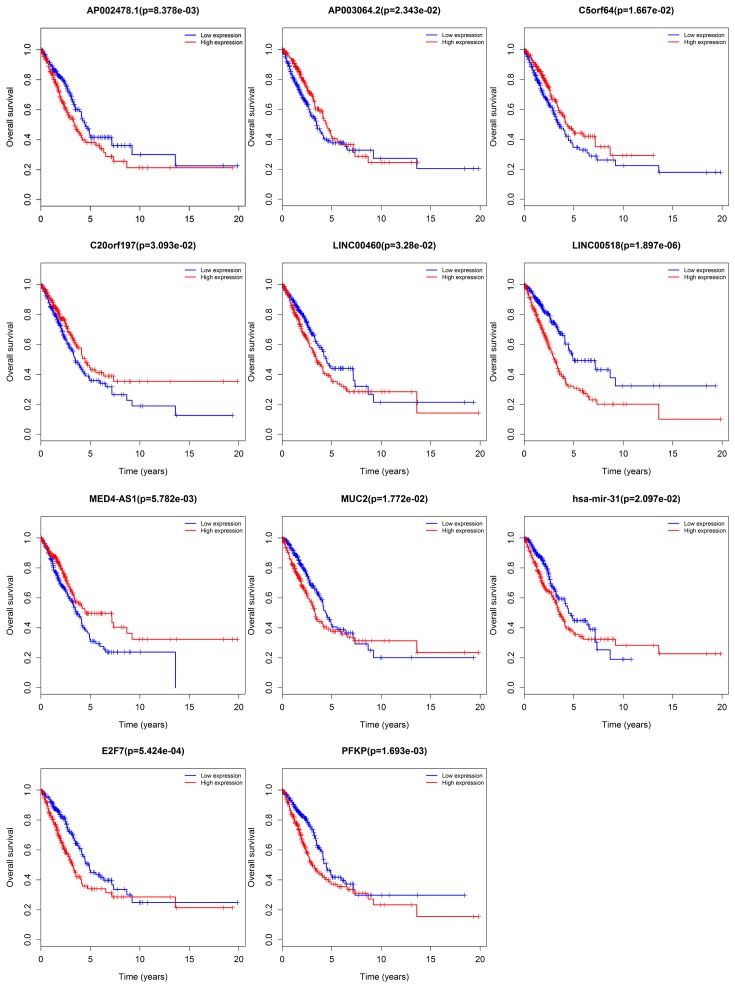
Kaplan–Meier survival curves and Log–rank for DElncRNAs, DEmiRNAs, and DEmRNAs as independent prognostic factors associated with OS in LUAD Horizontal axis is OS time (years) and vertical axis stands for survival function.

**Figure 6:**
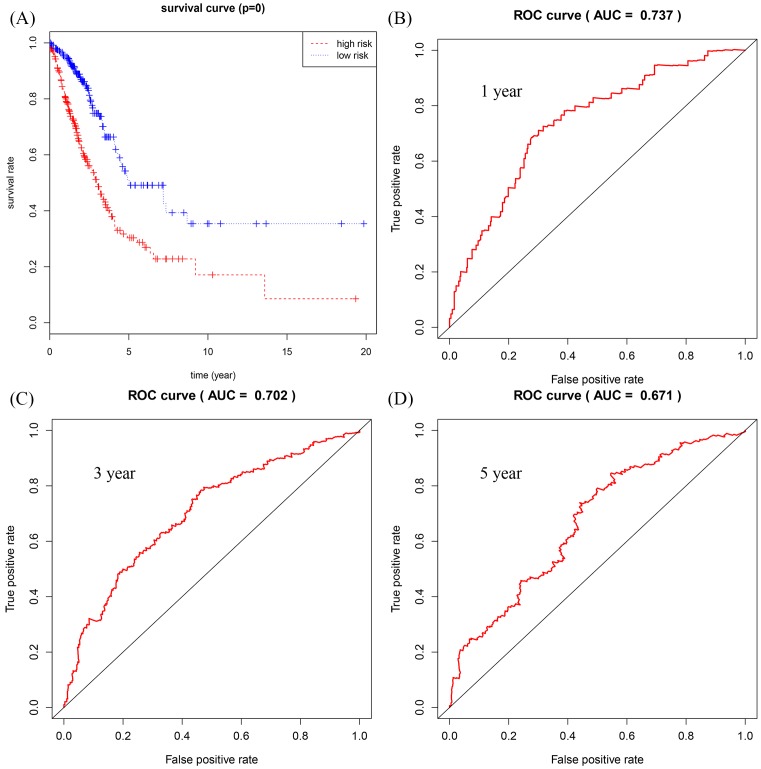
Construction of survival risk score system based on lncRNAs signature Cox regression analysis for survival prediction by the 8-lncRNA signature screened from 22 most significantly expressed lncRNAs with *P* < 0.01. (**A**) The survival curve of patients with high risk and low risk. (**B**–**D**) The ROC curve in 1, 3, 5 years with AUC value.

**Figure 7:**
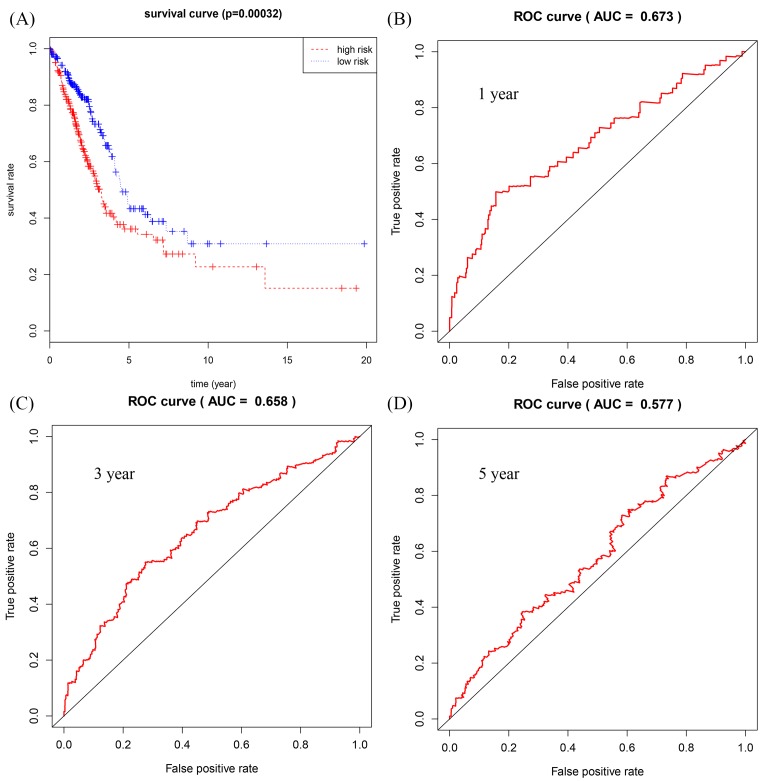
Construction of survival risk score system based on mRNAs signature Cox regression analysis for survival prediction by the 2-mRNA signature screened from 9 most significantly expressed mRNAs with *P* < 0.01. (**A**) The survival curve of patients with high risk and low risk. (**B**–**D**) The ROC curve in 1, 3, 5 years with AUC value.

**Figure 8:**
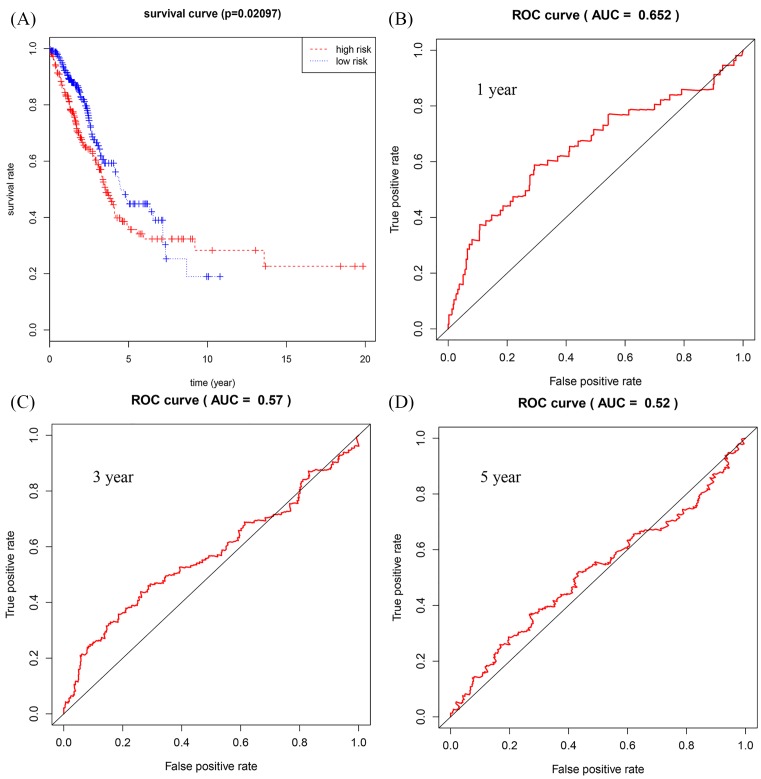
Construction of survival risk score system based on miRNAs signature Cox regression analysis for survival prediction by the 1-miRNA signature with *P* < 0.01. (**A**) The survival curve of patients with high risk and low risk. (**B**–**D**) The ROC curve in 1, 3, 5 years with AUC value.

The relationship between DElncRNAs, DEmiRNAs, and DEmRNAs with OS in the ceRNA network was studied in detail. With *P* < 0.05 as the standard, a total of 22 lncRNAs, 12 mRNAs, and 1 miRNA were identified to be associated with prognosis. These significant survival-related lncRNAs, miRNAs, and mRNAs were further submitted to univariate and multivariate Cox regression analyses to determine independent prognostic factors for LUAD. Subsequently, 12 lncRNAs, 9 mRNAs, and 1 miRNA were obtained using *P* < 0.01 as the initial screening criteria for univariate Cox regression analysis. The results were shown in Table 1. Next, we constructed the multivariate regression models with the three RNAs based on the univariate results. The three prognostic signatures contained 8 lncRNAs (*AP002478.1, AP003064.2, C5orf64, C20orf197, LINC00460, LINC00518, MED4-AS1, MUC2*), 2 mRNAs (*E2F7, PFKP*), and 1 miRNA (*hsa-mir-31*), and the relevant survival curves were showed in Figure 5. Using three signatures, we calculated the risk score for each patient separately and ranked them according to the increased risk score. According to the median risk score, LUAD patients were divided into high-risk and low-risk groups. K-M analysis showed significant differences of OS between high-risk and low-risk groups in all three models (Figures 6–8A). Our study predicted 1-, 3-, and 5-year survival rate accurately for LUAD patients. The time-dependent receiver operating characteristic (ROC) curves for lncRNAs (Figure 6B–D), mRNAs (Figure 7B–D), and miRNA (Figure 8B–D) signature have area under curve (AUC) values higher than 0.5. Among them, the PI of lncRNA showed good prognostic ability, which suggests that the prognostic model constructed by 8 prognostic lncRNA may be a good predictor of LUAD.

**Table 1:** Univariate Cox analysis of prognosis-associated RNAs in a ceRNA network of LUAD

RNA	HR	CI	*P*-value	Expression
**lncRNA**				
C20orf197	0.86	0.8–0.93	0.000160092	Up
C5orf64	0.81	0.7–0.95	0.007033541	Down
LINC00518	1.22	1.12–1.33	3.09E-06	Up
MUC2	1.08	1.03–1.13	0.003035376	Up
UCA1	1.09	1.03–1.14	0.001014659	Up
LINC00337	1.12	1.04–1.22	0.005081888	Up
MED4-AS1	0.82	0.71–0.94	0.004836856	Down
LINC00460	1.09	1.03–1.15	0.002507511	Up
SYNPR-AS1	0.90	0.85–0.97	0.00294044	Down
NAV2-AS2	0.84	0.75–0.94	0.003476934	Up
AP003064.2	0.87	0.78–0.96	0.007660574	Down
AP002478.1	1.11	1.04–1.18	0.00086024	Up
**mRNA**				
PFKP	1.28	1.13–1.45	0.000118643	Up
CLSPN	1.19	1.07–1.33	0.001424106	Up
RS1	0.87	0.79–0.96	0.00391744	Down
CCNE1	1.17	1.06–1.3	0.0018306	Up
CCNB1	1.32	1.16–1.49	1.28E-05	Up
KIF23	1.25	1.12–1.4	6.99E-05	Up
CEP55	1.24	1.11–1.38	0.000168944	Up
CHEK1	1.33	1.16–1.52	3.67E-05	Up
E2F7	1.25	1.13–1.39	1.14E-05	Up
**miRNA**				
hsa-mir-31	1.10	1.04–1.17	1.04–1.17	Up

Notes: ceRNA, competing endogenous RNA; LUAD, lung adenocarcinom; HR, hazard ratio; CI, confidence interval.

## Discussion

Lung adenocarcinoma is a multifactorial disease with high heterogeneity and mixed genetic factors. Most patients with LUAD are diagnosed in the advanced stage and have missed the opportunity for surgery [26]. Despite the improvement of lung cancer diagnosis, surgical techniques, and new drug treatments, the survival rate is still at a lower level [27]. With recent advancements, we are moving to a new era of personalized target therapies. It is becoming increasingly clear that many complex diseases, especially cancer, can rarely be attributed to a single genomic variation [28]. Therefore, it is very important to develop genomic detection of lung adenocarcinoma and predict the survival rate of patients according to their individual characteristics. A growing body of experimental evidence suggests that lncRNA plays an important role in many biological processes and, as a ceRNA, its involved in the occurrence and development of LUAD and can become a potential diagnostic and prognostic marker [29,30]. The ceRNA hypothesis reveals a new mechanism of RNA interaction and provides important clues and theoretical guidance for further understanding the tumorigenesis mechanism [12,15,31]. However, the expression pattern of ceRNA against LUAD and its prognostic value have not been thoroughly studied. In addition, compared with single-gene analysis, the prognostic characteristics based on multiple genes can help to predict the survival rate more accurately.

In our study, we identified DElncRNAs, DEmRNAs, and DEmiRNAs according to a large number of LUAD tissue samples from TCGA database, and constructed a ceRNA network, based on which we developed a genome-wide prognosis model. Our study has a deeper understanding of LUAD overall and also provides a new dimension for predicting the prognosis of individual patients with LUAD. In order to further study the potential functions of ceRNA networks, we performed GO and KEGG functional enrichment on mRNAs that were abnormally expressed in the network. The GO analysis revealed that the function of the aberrantly expressed mRNAs mainly concentrates on the nucleus, centrosome, and heterochromatin, which could participate in DNA replication and damage detection, and regulate the activity of cyclin-dependent protein serine/threonine kinases. Through KEGG pathway analysis, we detected a number of cancer-related pathways, including the p53 signaling pathway, the PI3K-Akt signaling pathway, and microRNAs in cancer. Studies have shown that *PPM1D* and *GADD45B* may regulate the progress of LUAD through the p53 signaling pathway [32], and that down-regulation of *CDKN2B-AS1* and *CDKN2A* may activate the p53 signaling pathway to promote lung cancer formation [33]. BOS-102 induces apoptosis and cell cycle arrest through reactive oxygen species (ROS)-mediated activation of the PI3K-Akt signaling pathway [34]. All these above views demonstrate that our ceRNA network reflects vital mechanisms of LUAD.

In addition, through the survival analysis of the RNAs in the network and the construction of the prognosis model, we found that the model constructed by 8 lncRNAs can be used as an independent predictor of OS in patients with LUAD. In the present study, there were eight prognostic lncRNAs and seven other lncRNAs besides *C20orf19* had shorter overall survival. To date, none of the lncRNAs *C20orf197, C5orf64, MED4*-AS1, or *AP003064.2* have been reported to have disease associations in any medical field. For the first time, we found that these four novel lncRNAs play a vital role in LUAD. Studies have shown that highly expressed *AP002478.1* has a poor prognosis in gastric cancer and hepatocellular carcinoma [35,36], and our study in LUAD also showed the same results, this suggests that *AP002478.1* plays an important role as a carcinogenic factor in many kinds of cancers. *LINC00518* was first discovered and developed as a non-invasive 2-gene molecule in the detection of cutaneous melanoma [37]. *LINC00518* was considered as a competitive endogenous RNA candidate for predicting breast cancer prognosis and could reduce multidrug resistance [25,38]. Its overexpression promotes paclitaxel resistance in prostate cancer by isolating *miR-216b-5*p [39]. MUC2 is the major mucin in the formation of intestinal epithelium [40]. Numerous studies have shown that the expression of MUC2 is related to the invasion and metastasis of various malignant tumors, including gastric cancer, gallbladder cancer, breast cancer, ovarian cancer, and lung cancer [41–45]. *LINC00460*, located on chromosome 13q33.2. has been shown to be up-regulated in cancers such as non-small cell lung cancer, esophageal squamous cell carcinoma, nasopharyngeal carcinoma, and thyroid carcinoma [46–49]. It can promote the migration and invasion of non-small cell lung cancer cells, induce epithelial–mesenchymal transition, and inhibit the growth of esophageal squamous cell carcinoma cells by regulating cell proliferation and apoptosis. These reports suggest that lncRNA can have a profound impact on the prognosis of LUAD, improve the accuracy of prognosis prediction, and emphasize the prognostic value of lncRNAs.

We built a genome-wide prognostic model based on a large number of samples from the TCGA database. However, several limitations must be noted. First, a longer follow-up time is needed to verify the reliability of our model. Second, these eight prognostic lncRNAs need to be validated experimentally, which will be carried out in our follow-up study.

## Conclusion

In conclusion, we identified prognostic biomarkers of lung adenocarcinoma based on competitive endogenous RNA network and Cox regression analysis, and constructed a model containing eight lncRNAs to reliably predict the prognosis of LUAD patients. Our research will contribute in further understanding the pathogenesis of LUAD and lay the foundation for future clinical research.

## Abbreviations

AUC: area under curve
BP: biological process
CC: cellular component
ceRNA: competing endogenous RNA
DElncRNA: differential expression of lncRNA
DEmiRNA: differential expression of miRNA
DEmRNA: differential expression of mRNA
GO: Gene ontology
KEGG: Kyoto Encyclopedia of Gene and Genome
LUAD: lung adenocarcinom
lncRNA: long non-coding RNA
MF: molecular function
miRNA: microRNA
mRNA: messenger RNA
OS: overall survival
TCGA: the cancer genome atlas
